# Use and experience of Italian healthcare professionals with aripiprazole once-monthly 400mg two-injection start initiation regimen in adult patients with schizophrenia

**DOI:** 10.1192/j.eurpsy.2025.2065

**Published:** 2025-08-26

**Authors:** C. Beckham, M. Yildirim, S. Pappa, K. Leopold, W. J. Cottam, J. Hickey, O. Rogerson, A. Fagiolini

**Affiliations:** 1 Otsuka Pharmaceutical Europe Ltd., Berkshire, United Kingdom; 2 H. Lundbeck A/S, Valby, Denmark; 3 Division of Psychiatry, Department of Brain Sciences, Imperial College London, London, United Kingdom; 4 Department of Psychiatry, Psychotherapy and Psychosomatics, Vivantes Hospital am Urban and Vivantes Hospital im Friedrichshain, Charite, Universitätsmedizin, Berlin; 5 Department of Psychiatry and Psychotherapy, Carl Gustav Carus University Hospital, Technische Universität Dresden, Dresden, Germany; 6 Real-World Evidence, OPEN Health, London, United Kingdom; 7 Università Di Siena, Siena, Italy

## Abstract

**Introduction:**

Aripiprazole once monthly 400mg (AOM400) is a long-acting injectable (LAI) available as a two-injection start initiation regimen (AOM400-TIS) for the maintenance treatment of adult patients with schizophrenia stabilised with oral aripiprazole.

**Objectives:**

This survey sought to explore HCPs’ perspectives and attitudes towards prescribing and/or administering AOM400-TIS according to the European label in clinical practice (including reasons for its use, potential benefits, and common barriers and/or concerns) across Europe.

**Methods:**

HCPs who had prescribed and/or administered the AOM400-TIS regimen to ≥3 patients with schizophrenia were invited to participate in an online survey. The survey was launched in two waves across the target countries (wave 1: Italy, Germany, United Kingdom; wave 2: Denmark, Italy, Sweden). Analysis was descriptive; data was collected between February 1–March 21, 2024 (wave 1) and September 16-October 28 (wave 2). Data from Italian HCPs are presented.

**Results:**

31 HCPs from the 1st wave and 64 from the 2nd wave completed the survey including psychiatrists (69%), psychiatric nurses (23%), community nurses (4%) and general practitioners/primary care practitioners (1%). HCPs estimated 30.0% (median; IQR: 20.0–50.0) of patients in their caseload were diagnosed with schizophrenia, and of these, 45.0% were treated with LAIs (median; IQR: 25.0–62.5). 47% of HCPs were primarily responsible for prescribing AOM400-TIS, 24% for administering it, and 28% were responsible for both. HCPs estimated that 44% of patients typically spent up to 14 days on oral aripiprazole prior to AOM400-TIS, with HCPs rating the severity of symptoms of patients initiated with AOM400-TIS as mild (22% of HCPs), moderate (68% of HCPs) and severe (40% of HCPs). The most common reasons for initiating AOM400-TIS after transitioning from oral aripiprazole were poor adherence (80%) and patient preference (49%), and the most reported goals for prescribing AOM400-TIS were to improve adherence (75%) and prevent relapses (69%). Common barriers to the use of AOM400-TIS were patient reluctance to receive two injections (39%), concerns about tolerability (24%), safety of administering a high dose in a single day (23%). Prior treatment adherence (54%) and efficacy (46%) were the most cited factors influencing prescribing of AOM400-TIS. Overall, HCPs “agreed”, or “strongly agreed”, that AOM400-TIS was easy to administer (81%) and that it had a similar safety/tolerability profile to the single injection start regimen (69%), while the majority were satisfied with patient outcomes with AOM400-TIS (83%).

**Conclusions:**

Overall, Italian HCPs with experience of using AOM400-TIS reported that it is easy to administer, well tolerated and improves treatment outcomes, while barriers to its use include patient reluctance and perceived safety concerns.

**Disclosure of Interest:**

C. Beckham Employee of: Clodagh Beckham is a full-time employee of Otsuka Pharmaceutical Europe Ltd., Berkshire, UK., M. Yildirim Employee of: Murat Yildirim is a full-time employee of H.Lundbeck A/S, Valby, Copenhagen., S. Pappa Grant / Research support from: Recordati, Janssen, Consultant of: Lundbeck, Janssen, Otsuka, Recordati, Rovi, Gedeon Richter, Sunovion., Speakers bureau of: Lundbeck, Janssen, Otsuka, Recordati, Rovi, Gedeon Richter, Sunovion., K. Leopold Grant / Research support from: Janssen, Otsuka, Consultant of: Boehringer Ingelheim, Lundbeck, Janssen, Otsuka Recordati, ROVI, Speakers bureau of: Boehringer Ingelheim, Lundbeck, Janssen, Otsuka Recordati, ROVI, W. Cottam Employee of: Will Cottam is a full-time employee of OPEN Health (London)., J. Hickey Employee of: Joe Hickey is a full-time employee of OPEN Health (London)., O. Rogerson Employee of: Olivia Rogerson is a full-time employee of OPEN Health (London)., A. Fagiolini Grant / Research support from: Angelini, Boehringer Ingelheim, Lundbeck, Janssen, Otsuka, Pfizer, Recordati, Viatris, Consultant of: Angelini, Boehringer Ingelheim, Idorsia, Italfarmaco, Lundbeck, Janssen, Medicamenta, Mylan, Otsuka, Pfizer, Recordati, Rovi, Sunovion, Teva, Viatris

